# Different Aspects of Self-Reported Quality of Life in 450 German Melanoma Survivors

**DOI:** 10.3390/cancers3022316

**Published:** 2011-05-11

**Authors:** Annika Waldmann, Sandra Nolte, Ron Pritzkuleit, Eckhard W. Breitbart, Alexander Katalinic

**Affiliations:** 1 Institute of Cancer Epidemiology (IKE e.V.), University of Luebeck, Ratzeburger Allee 160 (Haus 50), Luebeck 23562, Germany; E-Mails: Ron.Pritzkuleit@krebsregister-sh.de (R.P.); Alexander.Katalinic@krebsregister-sh.de (A.K.); 2 Association of Dermatological Prevention (ADP e.V.), Cremon 11, Hamburg 20457, Germany; E-Mails: sandra.nolte@web.de (S.N.); Eckhard.Breitbart@elbekliniken.de (E.W.B.); 3 Deakin University, 221 Burwood Highway, Burwood, VIC 3125, Australia; 4 Center of Dermatology, Am Krankenhaus 1, Buxtehude 21614, Germany

**Keywords:** skin neoplasms, melanoma, quality of life, population based, health care survey

## Abstract

The present study was aimed at assessing quality of life (QoL) in a total of 450 melanoma patients who filled out the EORTC QLQ-C30 (Q1; 15 months post diagnosis) as part of the OVIS Study. Follow-up questionnaires (Q2) were administered two years after Q1. The analyses presented herein were based on the following assumptions: QoL of melanoma patients is worse than that of a German reference population. Further, both tumor location and tumor stage have an influence on self-reported QoL, with patients with tumors located on face, head, neck, and advanced tumor stage (T3/T4) reporting the worst QoL levels. Finally, patients' QoL improves over time based on the theory of disease adaptation. In contrast to the above assumptions, with the exception of global health/QoL scores, differences between OVIS and the reference population were below the minimal clinical important difference of ten points. Furthermore, no clinically meaningful differences were found between patients after stratifying our data by tumor location and tumor stage. Finally, no clinically relevant changes were seen between Q1 and Q2 across all scales of the EORTC QLQ-C30. However, when data were stratified by patients with stable disease versus those with progression, clinically relevant differences were found between Q1 and Q2 predominantly in women in the latter group regarding emotional function, insomnia, dyspnoea, and fatigue. The lack of clinically meaningful differences across strata (tumor location; tumor stage), time, and patients compared to a reference population is surprising. However, it is possible that the instrument used, a generic QoL instrument, is generally not sensitive enough to detect differences in melanoma patients. Our findings may further be explained by the fact that all patients included in our sample had been diagnosed well before Q1, *i.e.*, main illness adaptation processes may have occurred before study entry.

## 1. Introduction

Rising incidence rates of malignant melanoma (MM) are of worldwide concern, in particular in the white population. Recent data from Germany show a three- to fourfold increase in age-standardized incidence rates from 1980 until today [1]. Current incidence rates for Germany are 12.6/100,000 for women and 11.9/100,000 for men (ASR World) [2]. They are comparable to incidence rates of other European countries, in particular those in northwestern Europe [1]. In contrast, incidence rates are much lower than those observed in Australia which are 32/100,000 for women and 52/100,000 for men (ASR World) [3] but much higher than those observed in less developed countries which have been reported to be less than 1/100,000 (ASR World) for both men and women [4].

While incidence rates continue to rise, melanoma mortality rates have been stable since the 1980s [1]. Today, 5-year survival from small MM, *i.e.*, ≤1 mm, is close to 100%. In contrast, less than 50% of German patients diagnosed with late stage MM (>4 mm) survive the 5-year period post diagnosis [5]. The relative increase in survival, *i.e.*, rising incidence while mortality rates are stable, is mainly due to improved early detection measures as melanoma treatment has not changed markedly in recent years. Today's treatment of MM mostly consists of surgery with or without sentinel lymph node or lymph node resection. Only a small group of patients with late stage tumors and metastases additionally receive chemotherapy and/or interferon therapy [6]. Once tumors are treated, melanoma patients, regardless of tumor stage, remain at increased risk for disease progression for many years [7, 8]. In view of this increased risk, a further aspect of melanoma treatment is patient counseling about UV-behavior. Hence, further consequences of MM are its impact on a patient's lifestyle as well as on his/her social and professional activities [9].

As survival from MM has improved in recent years, patient-reported outcomes such as quality of life (QoL) have become an important aspect of cancer research. An increasing number of studies address QoL issues. However, while many studies can be found for cancer sites such as breast, prostate, and colorectal cancer, only little is known about the long-term effects of skin cancer on patients' QoL. The few studies published mainly focus on populations from specialized melanoma centers or specialized cancer centers that received additional therapy during short periods of time. Hence, the impact of skin cancer on long-term survivors from the general melanoma population is not well documented [10]. Further, comparisons between melanoma patients and the general population are still scarce. Only recently, a cross-sectional Dutch study on QoL in melanoma patients was published showing that melanoma survivors did not report diminished QoL compared with an age- and sex-matched sample from the general population [11].

For this reason, we aimed at investigating QoL in melanoma patients. In particular, it was explored whether tumor location and tumor stage had an impact on self-reported data, and whether patients showed changes in QoL over a period of two years. We also explored whether differences existed between melanoma patients and a general population. The analyses were based on the following assumptions: QoL of melanoma patients is worse than that of a German reference population. Further, both tumor location and tumor stage have an influence on self-report QoL, with patients with tumors located on face, head, neck, and advanced tumor stage (T3/T4) reporting worst QoL levels. Finally, patients' QoL improves over time based on the theory of disease adaptation.

## 2. Results and Discussion

A total of 1,503 patients with melanoma diagnosis between January 2002 and June 2004 were notified to the Cancer Registry of Schleswig-Holstein and met the inclusion criteria. Thereof, 741 patients could not be contacted as the notification only included a pseudonym but no information on name or postal address that was needed for contact (=“non-eligible patients”). The remaining 762 patients were contacted, of whom a total of 450 provided questionnaire data at both Q1 and Q2 (=“participants”). Those who did not provide data either at Q1 (n = 154) or at Q2 (n = 158) are considered as “non-respondents”. While basic information on the three cohorts can be found in Table 1, the main analyses of the present study were based on the 450 respondents.

### 2.1. Baseline Characteristics

Data in Table 1 indicates that the OVIS participants are a representative sample of Schleswig-Holstein's melanoma patients although they represent less than one third of all registry cases.

Of all 450 respondents, 46.2% were female. Mean age at diagnosis was 56 years, with women being, on average, five years younger than men. Compared to women, men were more likely to be of high social status and have a spouse (Table 1). OVIS participants were younger at diagnosis compared to the total cohort of all Schleswig-Holstein melanoma patients as registered in the state's cancer registry where men have a median age of 63 years and women have a median age of 57 years at diagnosis of MM (data not shown). This can be attributed to the fact that DCO-cases (death certification only notification) and patients with an anonymous notification (“non-eligible patients”), which are generally older patients, were not eligible to participate in the OVIS Study [12]. The observed age difference between OVIS patients and all Schleswig-Holstein's MM patients may lead to a slight overestimation of the QoL of the total population of MM patients by means of OVIS data, as our data indicate an inverse association between QoL and age of the patient, *i.e.*, lower function and more symptoms are reported with increasing age (data not shown).

In the total OVIS group, nearly half of the tumors were located on patients' arms or legs. This is mainly due to the high proportion of tumors located on extremities in women, while the torso was the main location for melanoma in men (Table 1). This distribution of MM locations follows a typical pattern as has been reported for Germany [13] and also in a recent SEER publication. SEER data of 28,793 men and 22,965 women showed that 18% of all MM were located on face, scalp, or neck, 34% on the trunk, and 43% on extremities [14]. Recent data from Germany indicated a very similar pattern to that described in SEER [15]. The tumor location is not only of prognostic value [13, 14, 16] but may also have an impact on QoL, as some tumors and corresponding scars are more visible than others.

Most patients in our study group were diagnosed with localized malignant melanoma; advanced, late stage tumors were rare (Table 1). However, five men, in contrast to none of the women, were diagnosed with positive lymph nodes. Again, the low proportion of OVIS patients with advanced tumors compared to all Schleswig-Holstein's MM patients may lead to a minor overestimation of self-reported QoL of the former, as we see a weak tendency towards worse QoL scores in the subgroup of patients with T3/T4 tumors. Further, the unstaged cases (Tx) might bias our results as one could assume that information is not missing (completely) at random. In a worst case scenario, all unstaged cases are assigned to the prognostically worse tumor stages T3 and T4. Then our QoL results would slightly overestimate the “true” QoL in the majority of the QoL scales. In a best case scenario all unstaged cases are regarded as Tis/T1 cases. Again, our results as shown in Table 2 would slightly overestimate the “true” QoL in the majority of the QoL scales. However, in both scenarios, changes on all QoL scores were below the minimal clinical important difference of 10 points.

### 2.2. QoL Data of MM Patients at Q1 and Comparison to the German Reference Population

Regarding the function scales at Q1, OVIS men scored highest on physical function with mean values above 90 points (median: 100), while they scored lowest on emotional function with mean values close to 80 points (median: 92; Table 3). With regard to symptom scales and items, OVIS men scored highest for insomnia (mean value close to 20; median value: zero), thus describing the highest level of impairment on this QoL domain. Furthermore, they scored lowest on nausea and vomiting (mean values: close to zero; median value: zero), indicating least problems with these symptoms.

OVIS women scored highest on physical function, too, with a mean value above 90 points (median: 100) and lowest on emotional function with a mean value slightly above 70 points (median: 83; Table 3). Highest levels of symptoms were also scored on insomnia (mean value slightly above 20 points; median: zero) and lowest levels on nausea and vomiting (mean value: close to zero; median: zero).

When comparing OVIS patients and the general population, scores on global health status/QoL were higher in the former group. However, in MM patients no clear general trend towards a higher or lower scoring of QoL on both function and symptom scales were seen. Furthermore, with the exception of the global health/QoL score, all other differences between OVIS and the German reference population were below the minimal clinical important difference of ten points and thus not clinically relevant (Table 3).

In contrast to our findings, Dutch melanoma patients with lymph node dissection reported regularly higher function scores and lower symptom scores compared to the German reference population for all EORTC QLQ-C30 presented herein. Furthermore, in addition to global health status/QoL, emotional functioning was also clinically relevantly higher compared to scores of the German reference population and thus of clinical relevance [18]. Similar to previous findings, it is difficult to interpret why Dutch patients report high QoL scores, which are similar to ratings of OVIS patients, but higher when compared to the German reference population. One reason may be found in cultural differences between German and Dutch people.

The lack of clinical relevant differences between OVIS patients/Dutch patients and the German reference population is difficult to interpret. It is conceivable that the questionnaire used, *i.e.*, a generic QoL instrument, may not be sensitive enough to detect disease-specific effects. That is, for melanoma patients, generic QoL may not be as affected as, for example, areas that are more specific to MM, such as sun avoidance behavior. Therefore, the combination of a generic and a disease-specific QoL instrument might be the most informative way of assessing QoL in MM patients [10, 11]. To our knowledge, only a few melanoma-specific instruments are available. Among others, one could use the English version of the FACT-M that focuses on physical domains in melanoma patients [18, 19]. However, the content of the FACT-M seems especially appropriate for patients with advanced MM and less for those from the general MM population [10]. When the OVIS Study was designed, we were not aware of a German version of a melanoma-specific QoL questionnaire. Thus, the lack of relevant findings might also be attributed to choosing the “wrong” QoL instrument.

Finally, it may be conceivable that there are no (measurable) differences between the two populations. This could be for two reasons: (1) OVIS patients may truly not be significantly affected by MM. Especially if diagnosed with less advanced MM, patients' subjective QoL may not be substantially affected, *i.e.*, they may truly feel similar, or indeed better, on global health status/QoL compared to their healthy counterparts. Alternatively; (2) some or most OVIS patients may have adapted to their new situation, hence experienced a response shift. Response shift is a phenomenon that has been described as a change in perspective as a result of a significant life event such as the diagnosis of disease or treatment thereof [20, 21]. Hence, by experiencing a response shift, they may have reconceptualized, recalibrated, and/or reprioritized questionnaire items of the EORTC QLQ-C30 in a way that comparison of scores, *i.e.*, in this case comparison with the German reference population, is limited. Given space constraints, further discussion on this topic can be found elsewhere [22].

### 2.3. The Influence of Tumor Location, Tumor stage, and Health Status at Q2 on Self-Reported QoL of Melanoma Patients

In addition to the comparison of QoL levels of OVIS patients with those of the general population, data were stratified by (a) tumor location (face, head, neck; torso; extremities; excluding 14 cases with unknown melanoma location; Table 4); (b) tumor stage (T1/Tis; T2; T3/T4; Tx; Table 2); and (c) health status at Q2 (stable health status; progression of disease or diagnosis of another tumor; Table 5). Thanks to additional information provided by physicians, the proportion of unstaged cases could be reduced to 20% (T-stage) and 50% (M-stage), respectively, as opposed to nearly 50% (T-stage) and 70% (M-stage), as usually observed at the Cancer Registry of Schleswig-Holstein for MM notifications at that time period. As the size of the subgroup with unknown tumor stage is still relatively high, it is included in the tables as a separate subgroup.

When data were stratified by tumor location and tumor stage, no clinically meaningful differences were found. Furthermore, there was no obvious trend towards one subgroup being less impaired than another, *i.e.*, substantial differences were not seen when data were stratified by tumor location or when data were stratified by tumor stage. The only exception was found in fatigue, with a tendency towards an increase with increasing tumor stage (difference of nine points in mean values; differences of 22 points in median values) (See above for a discussion of bias due to unstaged cases).

The lack of clinically meaningful differences was surprising, and contrary to our assumptions, since we know from studies on e.g., breast cancer and prostate cancer that tumor stage and the resulting differences in treatment with variances in aggressiveness have an impact on generic [23] and disease-specific QoL [24]. However, findings for MM patients are less clear. While some studies show an impact of tumor stage and therapy on QoL [24-26], others do not find significant differences [27]. One possible explanation for the difference regarding fatigue scores seen for the tumor stage subgroups might be the long-term effects of chemotherapy which is given in case of more advanced disease but not in localized MM. Further, the lack of differences for the remaining symptom and function scales might be explained by the advantageous stage distribution in OVIS patients and according therapy options. That is, for more than 75% of patients with melanoma, local surgical excision is an adequate therapy [10]. In contrast, the lack of significant differences in the total cohort and the group of patients with stable health status could also be a measurement artifact. As discussed above, it might be the case that a generic QoL instrument was not able to capture effects on QoL in MM patients. Currently, an EORTC QoL module for melanoma is underway which, compared to the generic EORTC QLQ-C30, should prove to be more sensitive to detect differences specific to melanoma patients. Hopefully, future studies using the disease-specific module will give more insight to the effect of tumor stage and tumor location on QoL in melanoma patients.

However, a total of 51 patients reported either progression of the disease (n = 38) or the development of another tumor (n = 13). They had a mean age of 59.7 years (SD: 16) at initial MM diagnosis. The stage distribution of the initial MM was comparable to that of all respondents (T1: 31.4%; T2: 25.5%; T3: 17.6%, T4: 2.0%, unstaged cases: 23.5%). When the QoL of these patients was compared to patients with stable health status, clinically relevant differences were found for the majority of the QoL scales (Table 5).

### 2.4. Change over Time

Three distinct periods of potential impact of MM on self-reported QoL during the melanoma experience can be described: diagnosis, treatment, and follow-up. In particular, diagnosis and the time period immediately following diagnosis, *i.e.*, treatment phase and acute survival phase, is often associated with decreases in QoL. Patients report pain, energy loss, and a negative impact of physical and emotional function on social activities. Patients also rate their overall health at a lower level. During the time of follow-up, symptoms often disappear [10, 28]. Our first data collection was conducted 1¼ years after diagnosis (Q1); the second contact was 3¼ years after diagnosis (Q2). Again, in view of the advantageous stage distribution in our cohort and given that more than 75% of MM patients are treated with local surgical excision [10], we have to assume that (a) respondents had already completed their (initial) treatment, thus being already in the ‘follow-up’ period at Q1; and that (b) no further intervention took place between Q1 and Q2 in the majority of the patients, *i.e.*, those with stable health status. Hence, major improvement of QoL due to illness-adaptation processes was not to be expected in our study. However, a total of 51 patients reported either progression of the disease (n = 38) or the development of another tumor (n = 13). In this particular cohort, patients reported a clinically meaningful change over time in some of the scales: emotional function (difference in means of women: −12.3), insomnia (difference in means of women: 10.2), dyspnoea (difference in means of women: 19.7), and fatigue (difference in means of women: 14.4; men: 10.4). In contrast, there were no clinically relevant differences between Q1 and Q2 across all scales of the EORTC QLQ-C30 when either only patients with stable disease or when the whole sample of 450 patients were considered (data not shown). This was also true for the subgroups of men and women (Table 3).

Further, it has to be considered that in melanoma patients—More often than in patients with other cancer sites—QoL is predominantly determined by psychological aspects and only to a lesser extent by (long-term) therapy-induced events [10, 27]. Unfortunately, we were not able to evaluate psychological aspects in our study and can therefore not prove whether this possible explanation for the lack of significant differences applies to our study participants.

## 3. Experimental

### 3.1. The OVIS Study

The data presented herein were collected as part of the OVIS Study (Onkologische Versorgung in Schleswig-Holstein; oncological care in the German state of Schleswig-Holstein). OVIS was aimed at evaluating medical care, long-term consequences, and QoL issues of patients with breast cancer, prostate cancer, or MM.

The OVIS Study is a population-based statewide cohort study of cancer patients with the following inclusion criteria: Age at diagnosis 18 to 85 years, primary tumor of the breast (ICD-10 C50), primary prostate cancer (ICD-10 C61) or a primary malignant melanoma of the skin (ICD-10 C43) notified to the Cancer Registry Schleswig-Holstein, Germany, between January 2002 and June 2004. Participants of the OVIS Study were recruited by means of cases that had been notified to the state's epidemiological (population-based) Cancer Registry. Every physician that diagnoses or treats melanoma patients in Schleswig-Holstein, Germany, has to notify the tumor cases to the state's registry. As melanoma diagnosis and treatment involves different disciplines, it is possible that more than one notification is available for a single patient. These notifications are then quality-assured and combined into a ‘best-of-information’ for each patient in the registry. Thus, the full information for each patient is only available after about ¾ to 1¼ years.

For the purpose of the present study, melanoma patients were contacted via mail at two different points in time. The first contact was 1¼ years and the second contact was 3¼ years after diagnosis and we refer to these time points as Q1 and Q2. Both study letters included a detailed description of the research project, a consent form, a questionnaire, and a reply-paid envelope. Non-respondents were sent up to two reminders in week 4 and week 8 after receiving the first mailing. If these attempts did not result in a response, vital status and/or address changes were checked at the local registration office.

Patients' questionnaires consisted of three parts. In the first part, questions regarding medical care were included (e.g., type of assessment, mode of therapy, diagnosis communication, utilization of alternative therapies, social support, satisfaction with medical care, *etc.*). In the second part, QoL was assessed (see below). In the last part of the questionnaire, socioeconomic and demographic data were collected (e.g., relationship status, housing situation, income, occupation, education, health insurance, *etc.*). Information on clinical data such as TNM, date of diagnosis, histology, morphology, and basic information on therapy was obtained through the cancer registry. In some cases, physicians identified as the main contact by the study participants provided additional data such as missing information on TNM, and more detailed information on therapy. For further information on the questionnaires refer to the website of the Cancer Registry Schleswig-Holstein [29] and navigate to “Projekte”—“Deutsche Krebshilfe—OVIS”.

The ethics committee of the University of Luebeck approved the study protocol. Participation in the OVIS Study was voluntary and written consent was obtained from all participants.

### 3.2. Quality of Life Assessment

QoL was assessed using the validated cancer-specific questionnaire QLQ-C30 of the European Organization for Research and Treatment of Cancer (EORTC). The EORTC QLQ-C30 is a patient self-rating questionnaire that consists of five function scales (*i.e.*, physical, role, social, emotional, and cognitive functions), three symptom scales (*i.e.*, fatigue, nausea/vomiting, and pain), and five single items assessing symptoms such as dyspnoea, insomnia, appetite loss, constipation, and diarrhea. A final item evaluates the perceived economic consequences of the disease. Furthermore, a global health status/QoL score can be computed. According to the EORTC scoring manual, all scores of the QLQ-C30 were transformed linearly so that all scales range from 0 to 100. In the function scales, higher scores represent a better level of functioning; in the symptom scales/items, higher scores are indicative of a higher level of symptomatology or problems [30].

To make comparisons with a German reference population, we used general population data that are available for the EORTC QLQ-C30. This population was selected at random. That is, a random-route-technique based on 216 sample points (random selection of street, house, flat, and target subject in the household) was used. The population consisted of 2,028 persons with a mean age of 49.4 years (SD: 17.2; range: 16–92), and 56 % were women [31]. The age structure of this reference population was later adjusted to the age structure of OVIS participants following recommendations from Hjermstad *et al.* [17].

### 3.3. Statistical Analyses

Data were analyzed using SPSS for Windows (version 17.0). Results are presented as means ± standard deviation (SD) for continuous variables and as relative frequencies for categorical variables. Since a number of QoL scores were skewed, medians (25th–75th percentiles) are given for these variables. Additionally, means (SD) are given to allow for comparison to QoL scores of the German reference population that are provided in means only.

Differences between QoL scores of the general population and the OVIS population, intergroup differences (T-stages, location) as well as differences between Q1 and Q2 scores are interpreted in a descriptive way as suggested by Osoba *et al.*, *i.e.*, a differences of ten or more points are considered as moderate clinically meaningful differences [32].

## 4. Conclusions

Our study is one of the first studies comparing self-report quality of life of melanoma patients with that of a general population. The representative sample of 450 German melanoma patients includes patients with small to late stage tumors. QoL data were collected at 15 months post diagnosis (Q1) and again after two years (Q2).

Contrary to our assumption, QoL of the overall melanoma cohort did not differ from that of the general population. One possible explanation for the lack of differences between the two groups may be that the majority of melanoma patients had already adjusted to their situation, *i.e.*, at 15 months post diagnosis it is possible that main disease adaptation processes and/or response shifts already occurred before the study started. Alternatively, it is also possible that the EORTC QLQ-C30, a generic QoL instrument, is not sensitive enough to measure QoL-related issues that are specific to a melanoma cohort, *i.e.*, the instrument may be inappropriate to differentiate between melanoma patients and a general population. Future studies in this area should therefore include melanoma-specific instruments, such as the EORTC QoL module for melanoma which is currently being developed. Compared to the generic instrument, the melanoma-specific module should prove to be more sensitive to detect potential differences between melanoma patients and the general population.

Furthermore, and contrary to our next assumption, no significant differences were found after stratifying our data by tumor location and tumor stage. This finding is again surprising and may be a further indication that the instrument used is not sufficiently sensitive for melanoma patients, *i.e.*, in this case, it may not have been sensitive enough to differentiate between subgroups with respect to tumor location and tumor stage.

Finally, our results indicate that clinically relevant changes did not occur between Q1 and Q2 across all scales of the EORTC QLQ-C30 both in the total cohort and the subgroup of patients with stable disease. However, a clinically meaningful decrease in emotional function and clinically relevant increases in insomnia, dyspnoea, and fatigue over time were found in patients with progression. While worsening is to be expected in the latter subgroup, we anticipated a positive change in patients with stable disease based on the theory of disease adaptation. However, no change was seen. One possible explanation is the fact that patients had already been diagnosed 15 months prior to Q1, *i.e.*, it is conceivable that potential adaptation processes may have occurred well before our study started.

Our study provides important insight into various aspects of self-reported quality of life of melanoma patients. While it is one of the most comprehensive QoL studies in this area to date, future research is essential, in particular studies including melanoma-specific instruments, to explore whether the application of a generic instrument is sensitive enough to measure aspects of QoL that may be specifically relevant to people affected by melanoma of the skin. Based on the results of our study, it appears that self-reported QoL of melanoma patients is generally stable across subgroups (tumor location, tumor stage), generally stable over time, somewhat affected by health status, and finally comparable to that of the general population.

## Figures and Tables

**Table 1.** Baseline characteristics of 450 patients with incident melanoma shown as absolute (relative) frequencies or means ± SD.

	**Participants**		**Non-Respondents**		**Non-Eligible (anonymous notification)**	

**Personal data**	**OVIS Men [n = 208]**	**OVIS Women [n = 242]**	**Men [n = 143]**	**Women [n = 169]**	**Men [n = 302]**	**Women [n = 439]**

Age at diagnosis [in years]	59.0 ± 13.8	53.9 ± 15.8	57.7 ± 16.8	53.3 ± 18.2	57.1 ± 15.2	52.7 ± 16.9

Social status 1						
low	24 (11.5)	25 (10.3)				
intermediate	123 (59.1)	165 (68.2)				
high	59 (28.4)	41 (16.9)				
unknown	2 (1.0)	11 (4.5)				

Having a spouse	193 (92.8)	176 (72.7)				

**Clinical data**						

T-category						
Tis	2 (1.0)	2 (0.8)	-	-	-	-
T1	70 (33.7)	73 (30.2)	37 (25.9)	61 (36.1)	93 (30.8)	137 (31.2)
T2	52 (25.0)	73 (30.2)	25 (17.5)	19 (11.2)	34 (11.3)	58 (13.2)
T3	36 (17.3)	34 (14.0)	24 (16.8)	24 (14.2)	25 (8.3)	31 (7.1)
T4	4 (1.9)	6 (2.5)	7 (4.9)	5 (3.0)	10 (3.3)	15 (3.4)
Tx (unknown)	44 (21.2)	54 (22.3)	50 (35.0)	60 (35.5)	140 (46.4)	198 (45.1)

N						
N0	100 (48.1)	127 (52.5)	51 (35.7)	71 (42.0)	91 (30.1)	129 (29.4)
N1	4 (1.9)	-	4 (2.8)	-	7 (2.3)	5 (1.1)
N2	1 (0.5)	-	3 (2.1)	2 (1.2)	3 (1.0)	1 (0.2)
Nx (unknown)	103 (49.5)	115 (47.5)	85 (59.4)	96 (56.8)	201 (66.6)	304 (69.2)

M						
M0	104 (50.0)	125 (51.7)	55 (38.5)	73 (43.2)	93 (30.8)	132 (30.1)
M1	-	-	-	-	11 (3.6)	4 (0.9)
Mx (unknown)	104 (50.0)	117 (48.3)	88 (61.5)	96 (56.8)	198 (65.6)	303 (69.0)

Location of tumor						
Face, Neck	40 (19.2)	25 (10.3)	11 (15.5)	11 (13.3)	35 (11.6)	41 (9.3)
Torso	101 (48.6)	57 (23.6)	34 (47.9)	19 (22.9)	144 (47.7)	100 (22.8)
Extremities	62 (29.8)	151 (62.4)	24 (33.8)	52 (62.7)	97 (32.1)	270 (61.5)
unknown	5 (2.4)	9 (3.7)	2 (2.8)	1 (1.2)	26 (8.6)	28 (6.4)

Tumor thickness according to Breslow [in mm] 2	1.35 ± 6.78	0.76 ± 0.81				

Clark level 2						
1	7 (3.4)	2 (0.8)				
2	30 (14.4)	42 (17.4)				
3	47 (22.6)	62 (25.6)				
4	28 (13.5)	19 (7.9)				
5	-	2 (0.8)				
unknown	96 (46.2)	115 (47.5)				

1 Information on both social and relationship status was derived from the questionnaires and is therefore only available for “participants”;

2 Information on tumor thickness and Clark level was obtained from physicians when patients gave their written consent to contact them and is therefore not available for “non-eligible” and “non-respondent” patients.

**Table 2.** Mean (SD) and median (25th–75th percentiles) EORTC QLQ-C30 scores of German melanoma patients at baseline (Q1; OVIS I) according to tumor stage.

	**T1/T *in situ* [n = 147]**	**T2 [n = 125]**	**T3/T4 [n = 80]**	**Unknown T-stage (Tx) [n = 98]**
Global health status/QoL	76.1 (22.4) 83.3 (66.7–91.7)	74.3 (22.6) 83.3 (58.3–91.7)	73.7 (21.1) 83.3 (58.3–83.3)	72.8 (22.7) 83.3 (62.5–83.3)
Physical functioning (revised)	93.6 (16.5) 100 (100–100)	92.4 (18.8) 100 (100–100)	92.0 (17.6) 100 (100–100)	92.1 (15.9) 100 (95–100)
Role functioning (revised)	87.6 (24.0) 100 (83.3–100)	89.6 (20.8) 100 (100–100)	82.7 (27.9) 100 (66.7–100)	86.3 (25.6) 100 (79.2–100)
Emotional functioning	76.3 (25.1) 83.3 (58.3–100)	76.1 (28.6) 86.1 (66.7–100)	75.7 (23.4) 83.3 (58.3–100)	77.2 (25.3) 83.3 (66.7–100)
Cognitive functioning	89.3 (20.1) 100 (83.3–100)	86.8 (22.7) 100 (75–100)	89.4 (19.5) 100 (83.3–100)	91.5 (17.6) 100 (83.3–100)
Social functioning	86.5 (24.1) 100 (83.3–100)	86.9 (26.0) 100 (83.3–100)	81.8 (27.2) 100 (66.7–100)	88.8 (21.4) 100 (83.3–100)
Fatigue	16.2 (22.5) 0 (0–22.2)	17.2 (28.8) 0 (0–22.2)	25.5 (28.5) 22.2 (0–33.3)	17.8 (25.4) 11.1 (0–22.2)
Nausea and vomiting	1.2 (6.2) 0 (0–0)	2.6 (9.5) 0 (0–0)	2.0 (8.2) 0 (0–0)	2.7 (10.5) 0 (0–0)
Pain	14.0 (25.1) 0 (0–16.7)	15.5 (28.6) 0 (0–16.7)	16.7 (27.7) 0 (0–33.3)	14.4 (24.9) 0 (0–20.8)
Dyspnoea	13.1 (25.7) 0 (0–8.3)	11.6 (25.8) 0 (0–0)	12.7 (26.6) 0 (0–0)	11.5 (25.3) 0 (0–0)
Insomnia	17.7 (30.5) 0 (0–33.3)	24.7 (35.2) 0 (0–33.3)	19.9 (29.7) 0 (0–33.3)	22.0 (31.5) 0 (0–33.3)
Appetite loss	3.1 (11.8) 0 (0–0)	4.9 (14.7) 0 (0–0)	6.5 (19.5) 0 (0–0)	5.0 (18.9) 0 (0–0)
Constipation	7.0 (20.2) 0 (0–0)	4.3 (13.6) 0 (0–0)	4.8 (15.1) 0 (0–0)	4.6 (13.5) 0 (0–0)
Diarrhea	5.7 (16.9) 0 (0–0)	7.5 (20.7) 0 (0–0)	6.6 (17.2) 0 (0–0)	5.7 (18.7) 0 (0–0)
Financial difficulties	5.8 (17.8) 0 (0–0)	6.3 (17.9) 0 (0–0)	7.3 (18.3) 0 (0–0)	2.8 (11.6) 0 (0–0)

**Table 3.** Mean (SD) and median (25th–75th percentiles) EORTC QLQ-C30 scores of German melanoma patients at baseline (Q1; OVIS I) and follow-up (Q2; OVIS II) and the German reference population.

	German Men*	**OVIS I-Men [n = 208]**	**OVIS II-Men [n = 208]**	German Women*	**OVIS I-Women [n = 242]**	**OVIS II-Women [n = 242]**
Global health status/QoL	66.7	75.2 (21.8) 83.3 (66.7–91.7)	76.8 (21.3) 83.3 (66.7–91.7)	66.1	73.9 (22.7) 83.3 (58.3–91.7)	74.7 (22.2) 83.3 (58.3–100)
Physical functioning (revised)	86.0	93.0 (17.1) 100 (100–100)	91.9 (20) 100 (100–100)	85.3	92.3 (17.2) 100 (100–100)	91.8 (18.9) 100 (100–100)
Role functioning (revised)	84.1	87.4 (24.4) 100 (83.3–100)	87.0 (25.0) 100 (83.3–100)	83.3	86.7 (24.3) 100 (83.3–100)	85.1 (26.2) 100 (66.7–100)
Emotional functioning	78.6	79.4 (24.4) 91.7 (66.7–100)	79.8 (23.0) 83.3 (66.7–100)	74.2	73.6 (26.7) 83.3 (58.3–100)	72.5 (29.0) 83.3 (58.3–100)
Cognitive functioning	87.6	89.4 (19.6) 100 (83.3–100)	86.8 (21.0) 100 (83.3–100)	87.4	88.8 (20.8) 100 (83.3–100)	87.9 (21.0) 100 (83.3–100)
Social functioning	87.0	87.7 (23.4) 100 (83.3–100)	90 (21.9) 100 (100–100)	87.7	85.0 (25.8) 100 (83.3–100)	86.7 (23.0) 100 (83.3–100)
Fatigue	12.7	16.3 (24.7) 0 (0–22.2)	18.6 (25.4) 11.1 (0–22.2)	20.0	20.4 (27.3) 11.1 (0–33.3)	22.7 (27.5) 11.1 (0–33.3)
Nausea and vomiting	1.9	1.2 (5.2) 0 (0–0)	1.8 (6.8) 0 (0–0)	3.4	2.8 (10.6) 0 (0–0)	2.5 (10.5) 0 (0–0)
Pain	16.8	12.8 (23.8) 0 (0–16.7)	11.4 (21.9) 0 (0–16.7)	18.1	17.0 (28.5) 0 (0–33.3)	17.6 (28.1) 0 (0–33.3)
Dyspnoea	9.8	12.7 (27.0) 0 (0–0)	13.5 (26.4) 0 (0–0)	9.7	11.9 (24.6) 0 (0–0)	13.5 (26.6) 0 (0–33.3)
Insomnia	16.4	18.1 (30) 0 (0–33.3)	21.7 (31.3) 0 (0–33.3)	20.5	23.5 (33.4) 0 (0–33.3)	25.1 (32.9) 0 (0–33.3)
Appetite loss	4.9	3.9 (14.6) 0 (0–0)	5.6 (16.6) 0 (0–0)	6.2	5.2 (16.9) 0 (0–0)	5.7 (18.1) 0 (0–0)
Constipation	3.5	6.0 (17.6) 0 (0–0)	7.8 (21.0) 0 (0–0)	4.5	4.8 (15.1) 0 (0–0)	8.1 (21.7) 0 (0–0)
Diarrhea	2.5	5.6 (17.2) 0 (0–0)	6.2 (16.4) 0 (0–0)	2.8	7.1 (19.4) 0 (0–0)	6.8 (18.7) 0 (0–0)
Financial difficulties	7.3	6.2 (17.6) 0 (0–0)	7.7 (19.9) 0 (0–0)	6.6	5.1 (16.1) 0 (0–0)	8.4 (21.7) 0 (0–0)

* The age structure of the German reference population was adjusted to the age structure of the OVIS patients at baseline (‘direct age-standardization’ as suggested by Hjermstad *et al.* [17]). Therefore, only mean values are presented.

**Table 4.** Mean (SD) and median (25th–75th percentiles) EORTC QLQ-C30 scores of German melanoma patients at baseline (Q1; OVIS I) according to tumor location.

	**Face, Neck [n = 65]**	**Torso [n = 158]**	**Extremities [n = 213]**
Global health status/QoL	75.4 (23.4) 83.3 (0–100)	73.6 (23.2) 83.3 (0–100)	74.3 (21.4) 83.3 (8.3–100)
Physical functioning (revised)	90.1 (20.8) 100 (20–100)	94.5 (15.3) 100 (0–100)	91.9 (17.5) 100 (0–100)
Role functioning (revised)	82.8 (30.3) 100 (0–100)	88.6 (22.3) 100 (0–100)	86.3 (24.3) 100 (0–100)
Emotional functioning	80.4 (24.0) 91.7 (8.3–100)	75.8 (26.9) 83.3 (0–100)	74.8 (25.8) 83.3 (0–100)
Cognitive functioning	88.0 (21.3) 100 (0–100)	88.6 (20.1) 100 (0–100)	89.3 (20.6) 100 (16.7–100)
Social functioning	83.3 (30.2) 100 (0–100)	86.8 (24.7) 100 (0–100)	86.4 (23.3) 100 (0–100)
Fatigue	17.8 (26.5) 0 (0–100)	16.3 (24.1) 0 (0–100)	20.9 (27.9) 11.1 (0–100)
Nausea and vomiting	1.9 (10.8) 0 (0–83.3)	1.9 (7.4) 0 (0–50)	2.3 (8.9) 0 (0–66.7)
Pain	16.1 (27.9) 0 (0–100)	13.8 (25.1) 0 (0–100)	16.4 (27.7) 0 (0–100)
Dyspnoea	15.1 (27.8) 0 (0–100)	11.2 (24.5) 0 (0–100)	12.1 (25.6) 0 (0–100)
Insomnia	18.0 (29.2) 0 (0–100)	20.6 (32.1) 0 (0–100)	22.4 (33.1) 0 (0–100)
Appetite loss	6.9 (22.5) 0 (0–100)	3.7 (13.0) 0 (0–66.7)	4.9 (15.9) 0 (0–100)
Constipation	5.8 (18.5) 0 (0–100)	5.7 (17.8) 0 (0–100)	5.1 (14.7) 0 (0–66.7)
Diarrhea	4.8 (15.8) 0 (0–66.7)	7.1 (20.2) 0 (0–100)	6.3 (17.9) 0 (0–100)
Financial difficulties	7.9 (23.0) 0 (0–100)	4.1 (13.3) 0 (0–100)	6.4 (17.4) 0 (0–100)

Note: 14 cases with unknown melanoma location were excluded from the analysis.

**Table 5.** Mean (SD) and median (25th–75th percentiles) EORTC QLQ-C30 scores of German melanoma patients at follow-up (Q2; OVIS II) according to health status (progression or new cancer diagnosis *vs.* stable health status).

	**OVIS II-Men with progression/new cancer diagnosis [n = 29]**	**OVIS II-Men with stable health status [n = 176]**	**OVIS II-Women with progression/new cancer diagnosis [n = 22]**	**OVIS II-Women with stable health status [n = 210]**
Global health status/QoL	67.0 (27.7) 75 (45.8–83.3)	78.9 (19.2) 83.3 (66.7–91.7)	61.5 (29.6) 66.7 (33.3–83.3)	76.9 (20) 83.3 (66.7–100)
Physical functioning (revised)	82.8 (31) 100 (70–100)	93.5 (17.1) 100 (100–100)	80.9 (27.2) 100 (60–100)	94 (16) 100 (100–100)
Role functioning (revised)	69.0 (40) 100 (33.3–100)	90.4 (19.1) 100 (83.3–100)	69.8 (31.4) 66.7 (50–100)	87.5 (24.2) 100 (83.3–100)
Emotional functioning	67.2 (29.3) 75 (45.8–95.8)	82.4 (20.7) 91.7 (75–100)	58.3 (30.8) 58.3 (33.3–85.4)	74.2 (28.7) 83.3 (58.3–100)
Cognitive functioning	78.7 (34.7) 100 (58.3–100)	93.3 (16.7) 100 (83.3–100)	82.6 (24.4) 91.7 (79.2–100)	89.6 (19.2) 100 (83.3–100)
Social functioning	73.6 (34.7) 100 (41.7–100)	93.3 (16.7) 100 (100–100)	68.2 (34.5) 66.7 (50–100)	88.9 (20.2) 100 (83.3–100)
Fatigue	32.6 (35.8) 16.7 (0–66.7)	16.1 (21.6) 0 (0–22.2)	45.5 (31.4) 44.4 (22.2–77.7)	18.6 (24.5) 11.1 (0–33.3)
Nausea and vomiting	5.7 (10.2) 0 (0–16.7)	1.2 (5.9) 0 (0–0)	8.3 (22.3) 0 (0–0)	1.7 (8.1) 0 (0–0)
Pain	17.2 (27.3) 0 (0–33.3)	9.6 (19.5) 0 (0–16.7)	31.8 (29.5) 33.3 (0–50)	14.8 (25.8) 0 (0–16.7)
Dyspnoea	20.7 (31.4) 0 (0–50)	11.9 (25.2) 0 (0–0)	36.4 (38.4) 33.3 (0–66.7)	9.6 (22.1) 0 (0–0)
Insomnia	34.5 (35.7) 33.3 (0–66.7)	19.6 (30.1) 0 (0–33.3)	39.4 (42) 33.3 (0–75)	22.3 (30.7) 0 (0–33.3)
Appetite loss	16.1 (29) 0 (0–33.3)	5.6 (16.6) 0 (0–0)	11.1 (26.5) 0 (0–0)	4.9 (16.5) 0 (0–0)
Constipation	6.0 (17.6) 0 (0–0)	3.4 (12) 0 (0–0)	14.3 (29) 0 (0–16.7)	6.2 (18.5) 0 (0–0)
Diarrhea	6.9 (18.6) 0 (0–0)	6.0 (16) 0 (0–0)	7.9 (18) 0 (0–0)	6.6 (18.7) 0 (0–0)
Financial difficulties	16.7 (29.4) 0 (0–33.3)	5.8 (16.3) 0 (0–0)	16.7 (26.7) 0 (0–33.3)	6.4 (19.1) 0 (0–0)
